# When the non‐sharing of therapeutic goals becomes the problem: The story of a consumer and his addiction to methadone

**DOI:** 10.1111/jpm.12793

**Published:** 2021-09-23

**Authors:** Elena Faccio, Ludovica Aquili, Anonymous, Michele Rocelli

**Affiliations:** ^1^ Department of Philosophy, Sociology, Education and Applied Psychology University of Padua Padua Italy

**Keywords:** drug addiction, drug therapy, goals sharing, methadone, negotiation of therapy, rehab centre

## Abstract

**What is known on the subject?:**

- It is well known that psychotropic drugs, besides having beneficial effects, may become a source of addiction.
- Drug therapy involving methadone is traditionally considered an *essential medicine* in the treatment for heroin dependence since it significantly reduces drug injecting and death rates associated with opioid dependence.

**What the paper adds to existing knowledge?:**

- This paper investigates a paradoxical situation: the use of methadone therapy to maintain a condition of addiction rather than to overcome it. The story is told jointly by the head of a rehab centre and a young man who has developed a methadone addiction and kept it hidden for years from the operators of the addiction centre, who supplied him with the substance.

**What are the implications for mental‐health nursing?:**

- The young man's story offers a key example which can be of interest not only for addiction centres but also for all mental‐health services that supply drugs as therapy. This study examines what happens when a person taken over by a service pursues goals that are in contrast to the service mission.
- Specific communication strategies have to be implemented to update and negotiate goals in continuity with the personal live project of the service user.
- This story is a warning to not rely on consolidated operational practices, ignoring the investigation of personal meanings and aims of those who experience them.

**Abstract:**

**Introduction**

Mental health services typically follow standardized intervention protocols and systematic operating practices.

**Aim/Question**

This paper explores what happens when a service relies exclusively on fixed practices rather than on the negotiation of objectives and the differentiation of procedures according to the needs of the user.

**Method**

The analysis of the narratives produced in first person by Oliver, a former substance user, emphasizes the need of constructing a therapeutic plan in close cooperation, promoting an user active role and strengthening his affiliation in the change process.

**Results and Discussion**

In this specific case, the involvement of the user meant the identification of the peculiar meanings he attributed to the “substance of use,” which paradoxically coincided with the “therapeutic substance” (methadone).

**Implications for Practice**

Oliver's story is an effective mirror for rethinking staff conduct when it assumes that the drug is therapeutic in itself, regardless of the way the person experiences and means it, it also offers a description of the interactive ways in which the relationship between a young person and service staff proved to be effectively therapeutic.

## 1 INTRODUCTION

### 1.1 Drug therapy involving methadone: What experience do consumers of “therapeutic substances” report?

Pharmacotherapy represents a necessary component for many effective treatments but it can also induce addiction and the border is thin between effectiveness and dependence (Nielsen et al., 2012). Many patients complain of withdrawal symptoms when they try to break off or reduce the dosage (Davies & Read, 2019; Read, 2020). With regard to pharmacotherapy for consumers of psychotropic substances, treatment of people with heroin‐abuse problems very often involves methadone for long‐term therapeutic purposes (Bargagli et al., 2005; Iudici et al., 2015, 2020, World Health Organization, 2006). Although both heroin and methadone are known for their potential risks of addiction, it is customary to give them different meanings: heroin is a drug while methadone is a therapeutic substance. To state this distinction as unquestionable may expose to the risk of overlooking the variety of substance users' experiences of addiction. Indeed, we cannot avoid the task of investigating the specific meanings that users attribute to “substances for use” and “therapeutic substances.” Nor can it be assumed that they correspond to those of the practitioners working within the addiction centre: In some cases, methadone is seen by users as an addictive substance; conversely, the service considers it as a therapeutic drug. In this regard, Järvinen and Miller (2010) defined *methadone treatment* as the consolidation of a new addiction.

From a more broader point of view, the social context configures the use of illegal drugs, including heroin, as a dangerous activity, while methadone is considered either as a way to escape the risks associated with the use of illegal opioids or as medical treatment (Frank, 2018) or as a hybrid substance: on the one side, an illicit substance, on the other, necessary for recovery (Notley et al., 2015). The subjective interpretations by the side of consumers may greatly differ. Methadone users define the substance as beneficial because it guarantees a stable and legitimate lifestyle, but they do not consider it a *treatment*, functional to a self‐change process (Frank, 2018). To all this, they must be added the implications related to the process of stigmatization: we should not forget the stigma associated with taking methadone. Indeed, the self‐change process would also be hampered by stigmatizing attributions that methadone consumers have received from others precisely because they are on methadone treatment (Conner & Rosen, 2008; Doukas, 2011; Earnshaw et al., 2013; Faccio, 2013). Indeed, paradoxically, the stigma associated with drug addiction also expands to those in recovery and under methadone therapy. Mental‐health practitioners have to be aware that taking methadone does not guarantee per see the therapeutic change. The literature has shown that the meaning that methadone takes on, and the benefits of it, may change according to the perspective to which attention is paid; on the side of the consumer, methadone use takes on a plurality of connotations: it is both a liberation from the incessant demands for money associated with heroin consumption and an aid to lead a more normal life, but it is also a dangerous and addictive drug. On the side of the medical staff, it is a medicine like any other (Harris & Rhodes, 2013; Järvinen, 2008). The risk of not revealing nor toning the reciprocal representations may evolve into a full‐blown failure, where the continuity of the relationship between user and service is not an indicator of the effectiveness of the path but the means of an exploitation.

Some research (Granerud & Toft, 2015; Holt, 2007) has shown that methadone‐maintenance programmes are often perceived by users as demotivating and humiliating, with little influence on the management of their treatment. They are also seen as not aligned interventions with the users' needs and tending to de‐empowering the users' agency capabilities.

In addition, the user often perceives the methadone‐based programme as extremely controlling, to the point that the staff no longer assumes the role of facilitator for the management of the addiction, but rather becomes the ones from whom a simultaneous use of heroin is hidden (Grønnestad & Sagvaag, 2016). Such mechanisms do not facilitate a process of change, which is the real aim of all therapy. A mutual involvement and, in general, a positive and equal relationship between the consumer and nurses and social workers have instead proved to be fundamental for the success of the treatment, thanks to the promotion of a dialogue process that guarantees a shared definition of objectives (Vanderplasschen et al., 2015), necessary for the promotion of a personalized treatment (Rance & Treloar, 2015).

### 1.2 Oliver's story

Oliver (fictitious name) was a 27‐year‐old Italian boy when he decided to turn from the SerD (Service for Pathological Addictions) to a rehab centre for drug addicts, aiming at definitely stopping the drug consumption, declared at first to be heroin. The stay period of eight months at the rehab centre, a residential facility, which took place in 2016, had been a good opportunity for Oliver to rethink his story and review it critically. In fact, many actions previously put in place when he has been attending the SerD had been aimed at maintaining the addiction rather than overcoming it. First of all, the decision to hide, in dealing with the SerD, all the personal information that would have revealed his personal plan. Worrying that the SerD operators had not realized the unforgivable misunderstanding for 4 years.

With the help of the therapist in charge of the centre (MR), and thanks to the participants in the therapeutic group, he started to address some issues of importance about his experience. The dialogue between Oliver and the head of the rehabilitation centre, here faithfully transcribed, addressed some important points and helps us understand which operational and relational practices made it possible for Oliver to conceal for a long time the real reason why he benefited from the SerD in total contrast with the objectives and mission of the service itself.

He contributed to this research by narrating autobiographical events and agreed to its publication in the hope that others may benefit from his experiences by enhancing reflexivity about practices whose painful effects should not be passed over in silence or underestimated.

Oliver is a coauthor and has approved all the comments. The story has been introduced by a quick review of the scientific literature (by LA) and analysed (by EF) to present practical implications for the clinical setting.

### 1.3 Oliver's experience at the SerD and in the rehab centre for drug addicts: Which projects do the versions we tell about us reveal?

(Oliver): One day I turn up at the SerD: “Hi, I'm a substance user, I'm using heroin, and I've tried methadone to try to get off it.” So I undergo a urine test, which confirms the presence of heroin and methadone, as well as cannabis. After a cognitive interview with the doctor in charge, I was prescribed methadone. In the following days, I have a series of appointments with the professionals of the service: the social worker and the psychologist, to assess the case and my family history; the doctor, to monitor the progress of the new drug I was prescribed; and finally the educator. Most of the interactions took place with the nursing staff, who, on a daily basis, received me in order to dispense the predetermined dose of methadone.

(MR): A brief comment from the point of view of the addiction centre's practitioner: Oliver entered the service based on a standardized access; the urine positive for heroin and methadone, the report about the boy's use and the request for methadone provided sufficient elements to undertake a methadone‐based substitution therapy.

(Oliver): Every day, I show up to take the therapy, having more and more fixed and continuous interactions with the nurses, sporadic and functional ones with the rest of the team. Urine tests were always negative for heroin use. I interacted with the service when necessary or when requested; I made myself available for scheduled interviews with various professionals. I gradually became a silent and stabilized user. This went on for 4 years. That was until I met a girl with whom I started a relationship, and together we planned a holiday abroad. Within this new project, methadone started to become a problem. I faced a stumbling block: the use I was making of the substance was perhaps turning into an addiction; it was time to think about taking it away. The desire to get rid of it matured. So I went to the SerD, submitting a request to be referred to a rehab centre. It was a very painful choice for me; it meant taking a leave from work, leaving home, dealing with the slander in the country about my disappearance. Anyway, it was the only way, because my primary need was to scale up therapy.

(MR): At that point, the SerD who had taken charge of Oliver contacted me and told me your story. You had been described as a young boy addicted to heroin but who had not used the substance for about 4 years, being under methadone substitution therapy, stabilized at 45 mg per day. The colleague added that you had required a specific intervention to get off methadone, as you were intimidated by the prospect of living without the replacement therapy, which “is typical for users in the withdrawal from heroin”; he also said that you had created a strong bond with methadone, even if only as an antidote to heroin. At that time, I was the head of the rehab centre. I invited you to come to the centre a few times to assess your compatibility with our programme, and I, too, was unaware of what methadone use could mean to you. At the very beginning, you told me the same story you had told to the SerD. At that point, I shifted the focus from the substance to its demand: the fear of being sick and the desire to be well. You feared the side effects of not taking the substance.

(Oliver): I had previously tried to get off the drug independently, but after the second or third day, I was so sick that I was forced to take it again. I needed to be in a protected place, without the possibility of using substances, to try to get off methadone.

(MR): You were very aware of what you were being given. Over time you had built up such a strong bond with the effects of methadone that you could describe every little change, both chemically and perceptually, as an alchemist. But something was not right. I had never seen anything like this before, with any other user of the facility. So I began to investigate what this substance, and the behaviours associated with taking it, meant to you. As a result of this reflexivity effort, you began to feel able to talk about yourself, about the difficulties you had in relationship with your parents, with a girlfriend who “tested” you a lot, with which you had fantasized that trip abroad so important, and that never took place, which had disturbed you so much that it was the source of your desire for heroin and opioids. You had started to open up. We then began to pay attention to the way you interacted with the substance. The aim was for me to enter into your world, to put the emphasis on the meaning the substance had for you rather than on the substance in itself, keeping ourselves away from the prejudices about the “typical drug‐taking experience.”

(Oliver): I started to perceive MR and the operators no longer as enemies but as allies. In addition because of the relationship that had been created, I “spilled the beans”: I confessed that I was actually a methadone user, not a heroin user. For me, methadone was not a substitution therapy but the substance of choice, the substance of use for which to call myself an *addict*. When the operators reduced the methadone, they reduced my substance of use. It was like saying to a cocaine addict, “Today we are taking away some cocaine compared to yesterday.” In the centre, I began to see the power and effects of a non‐judgmental environment. Whatever I said, the interactions between me and the staff would not have changed; the roles would not have changed. The staff would not have felt mocked, used or justified in treating me in another way. This allowed something to change. I was ready to tell a new story. At the SerD, I sometimes went to interview with the educator, other times with the psychologist. The SerD had a complete overview of my family, work and management issues. In addition, I also used the SerD as a situation where I could communicate with someone, confront myself, vent, tell how my life was going. But the service had for me not only the function of supporting me, but, and this is the main one, also that of providing me with the substance to use. I had managed to hide my true intentions from the SerD by adapting to what the SerD thought of me—namely that I was addicted to heroin. To do this, I used what I knew to be the “typical sayings” of the heroin user. Initially, I used the rhetoric of heroin craving (i.e. that methadone “didn't cover me enough,” meaning that it did not take away my desire to use heroin, to get me to up my therapy). Then, I simply continued to comply, to follow what the service told me to do.

(MR): You had adhered to a practice and had managed it in order to take your own advantage. You understood that methadone, within the SerD, had the one and only meaning of a therapeutic substance. You had adhered to the meanings that others had given. Your personal meanings and plans had not been explored. Everything had been well planned: the intake of a small dose of heroin happened just sometimes, enough to legitimize the demand for methadone, and among the benefits of taking methadone, let us not forget that it is free, “legal” and not very dangerous when it comes to the risk of overdose. Your personal use of methadone was totally different from the social mandate of the service to which you had turned. Paradoxically, we could say that the SerD had been your drug dealer. You had subverted the mission of the service, and service practitioners had remained unaware of the role that you had assigned to the SerD—namely that of provider for intoxication and not detoxification. You and the SerD shared the maintenance mandate but not the reason why you met: the former with the aim of finding the intoxicating substance; the latter with the opposite attempt to detoxify the person. The relationship with you changed when I realized that I was dealing with a person whose need was to detoxify with the same substance of use. Your movements in managing the substance or in agreeing with us operators on a practice of reducing methadone therapy were exponentially more difficult than those of all the other users. This led me to ask the question: “Why were you struggling so much?” Taking off one milligram was already a cause for concern because of the effects that could result. You were, compared to others, much more alert to the bodily changes involved in lowering the therapy, a sensitivity usually found with the substance of abuse, not with methadone.

(Oliver): I started to talk about my needs when I started to perceive the relationship with practitioners as solid, based on the search for common goals. I knew that keeping the secrecy of my situation would not be an effective strategy. I then decided to involve others in my “real” story, within the project I wanted to carry out. This was possible because I did not feel trapped in telling it.

(MR): From that moment I, but also you, started to pay more attention to the fact that you were noticing with a particular emphasis the moment when the methadone bottles were thrown away, that you had to stay away from the special waste bin, or that you were paying attention to how many drops were left in the bottle, whether it was emptied or not, things that the other guys overlooked. We started paying attention to a series of practices that were very important for you: the fact that you wanted to steal from the bin all the drops of methadone left at the bottom of each bottle, or the fact that you wanted to eat little in order to feel more intensely the effect of the substance and to wait to eat after 2 p.m. for the administration of methadone.

(Oliver): What allowed me to feel capable of managing the situation, and what made me want to be guided, was the possibility of constructing the aim together, without anyone having to adhere (or pretend to adhere) to the other's. My request, for example, to stop the reduction of the therapy (I never asked to increase it), in virtue of a tranquillity that was a bit wavering, was not perceived by the person in charge as a desire of evasion or exaggeration but as a desire on my part to use the substance to balance my moods.

### 1.4 Issues of importance

Oliver's story invites a reflection on the risks and implications associated with the repetition of crystallized practices and offers suggestions for rethinking the interactions between a service staff and the user, in view of the design of an intervention. Oliver has proposed different stories to drug services by virtue of the definition of different projects and needs. In addition, as he placed them in different contexts, these stories generated new possibilities.

The first story, the one told at SerD, was based on Oliver's need to find his favourite substance of use, methadone. The strategy put in place was therefore to impersonate the role of the perfect drug addict and to take on board the typical, shared and therefore stereotypical discourses associated with this role (i.e. to present oneself as a heroin user and, at the same time, as motivated to free oneself from this addiction, through the confirmation of the analyses and the rhetoric of the heroin desire). If a service indulges in the repetition of predefined practices, it risks explaining the other according to what it believes this is (i.e. the typical drug addict) instead of referring to what the person brings to it as personal and peculiar. According to the rules of that game, Oliver's coverage strategies were effective (Goffman, 1963). This stereotypical way of relating to users allowed Oliver to hide among established practices. At the same time, it prevented the service from questioning Oliver's unique experience. As the years passed, it became increasingly difficult for Oliver to reveal the real story since the “character” he played had to be coherent with the past request. Oliver's fear was that the service would judge him negatively for the lie he had told.

The incompatibility of two experiences—the use of the substance and a trip abroad—represented for Oliver a problem that required management according to new strategies and, specifically, through inclusion in a different, more protected context, such as the rehab centre. This new possibility allowed Oliver to tell a new story. We want to focus attention on this relationship—whether it was Oliver who made it possible to re‐narrate himself through the definition of a new need or whether it was thanks to the relationship with the operators, to their willingness to align themselves with the plot of the story brought by the person, that the personal meaning attributed to methadone was revealed.

What was decisive was the willingness of the operators to pay attention to Oliver's specific reactions to the management of the substance of use, using comparisons with the management methods of other consumers, with the intention of approaching the peculiar experience from what made it different and, therefore, personal. A non‐judgmental context, able to configure the new version of Oliver not as evidence of the previous deception but starting from the resources and possibilities opened up by virtue of the staff's knowledge of its demands, was essential to allow such sharing. What was successful was, firstly, the attention that the team paid to the way in which the boy interacted with the substance, to the way in which he spoke about it, and secondly, the curiosity of the team to know Oliver's world, to know the meaning that the substance had for him, the purposes for which it was used.

### 1.5 Collaborative writing

From a methodological point of view, we chose the collaborative writing method (Serpa et al., 2017), in which researchers/therapists support participants in writing about their experiences of suffering. In this way, the researchers take on the role of coauthors who help the participants organize their own stories. The distance between the researcher (MR) and the user (Oliver), which is traditionally valued in scientific research, is rejected in favour of the realization of an intersubjective game (Serpa et al., 2019). Once again, the interaction between operator and user is privileged: by collaborating, on an equal level, in the production of knowledge, MR and Oliver have produced a story that is the result of the interaction of their different voices and perspectives (Clark, 2014). Furthermore, this mode of presentation allows mental‐health services, firstly, to capture what the user reports as successful or unsuccessful from a therapeutic perspective, and secondly, to capture, from the therapist's own words, how such therapeutic success was achieved.

### 1.6 Conclusions

Participation and the sharing of intentions in a project of change by a person addicted to substances are necessary conditions to promote a sense of effectiveness recognized by all the actors involved. Oliver's story confronts the many risks of depersonalization of projects that arise when consolidated practices are proposed, within a service aimed at a generalized user base, such as that in a SerD (Faccio et al., 2017; Romaioli, & Faccio, 2012).

If a service does not propose to share the objectives with the user, the only possibility offered to the latter is adherence to the context and, at the same time, privatization of the aims (Faccio et al., 2020). The SerD can thus become a place for intoxication rather than detoxification; staff can become an obstacle to be circumvented rather than a resource to be used; a programme can become a maintenance of a career rather than a transformative event.

Oliver's experience teaches something very pragmatic to be made operative in interactions involving the user and the staff of a service.

Oliver interacted constantly with the service nurses, who acted as privileged observers of the boy's movements in the SerD. If exploited, these interactions would have proved crucial in exploring Oliver's story and opening up a space for joint action. This happened in the rehab centre, where the establishment of a personal relationship, capable of lowering the “filters” of what could or could not be said, was a fundamental step to open such a space for action.

In the relationship with Oliver, a keystone was shared, where the perception of the absence of judgement allowed new interactions to open up, aimed at discovering intentions, desires and curiosities of a story in such a way as to not constrain it within a label emanating from a reductionist view of substance use. We went a little further, where he allowed us to enter; we asked him what his goals were, what the substance allowed him to do, what value it had for him. Our curiosity was to learn about the unique interaction between Oliver and the substance of use.

Addiction services have the privilege to participate in the everyday lives of their users; the interactive knowledge of personal stories, not labels (“drug addict”), allows a service to personalize interventions (Faccio, 2011; Faccio et al., 2019). What worked with Oliver was, on the one hand, proposing him not to join our project in favour of building a programme together, taking into account his needs, inclinations, and concerns. It is the service that goes to the user, and not the other way around. On the contrary, it was successful because the professionals were welcoming and non‐judgmental, according to Oliver, unlike how the interactions were initially structured. Oliver managed to discover new spaces of action in the helpful relationships with professionals, transforming the rehab centre from a space chosen by him to a space agreed upon together (Faccio et al., 2018).

In the end, Oliver proposed a risk inherent in the practices of the services, that of losing the personalization of the treatments in the intervention protocols. Regulation of each service's practices on established working methods may help professionals manage as many cases as possible, but they must be balanced in the course of taking charge of each individual. People access addiction services with very different intentions, needs and objectives, sometimes unknown to the service and sometimes even in antithesis with the purpose for which the service was designed. Questioning the role and the part requested in the “play” allows both to be faithful to the mandate of the service and to be part of the project of change, rather than maintenance, requested by the user.

## CONFLICT OF INTEREST

The authors declared no conflict of interest.

## AUTHOR CONTRIBUTIONS

The design of the manuscript was made by the first author (EF) in order to extract practical implications for the clinical setting. The story has been introduced by a quick review of the scientific literature and analysed by the second author (LA). Oliver was interviewed by the fourth author (MR), who also concepted the idea at the base of this paper.

## ETHICAL STATEMENT

An academic ethics committee approved the study. Authors of these narratives of lived experience ensure that there is a genuine and equal collaboration and that the contextualization or analysis avoids any interpretation of someone else's experience that has not been validated by the anonymous author. Anonymity is maintained in discussing the service and the staff.

## Data Availability

The data that support the findings of this study are available on request from the corresponding author. The data are not publicly available due to privacy or ethical restrictions.
